# *Mycobacterium tuberculosis* latent infection in healthcare students: systematic review of prevalence

**DOI:** 10.1590/1980-220X-REEUSP-2023-0238en

**Published:** 2024-03-11

**Authors:** Renata Silva de Lima, Rodrigo Nogueira da Silva, Suzana Rosa André, Ana Kedma Correa Pinheiro, Ana Inês Sousa, Ingrid Fabiane Santos da Silva, Juliano dos Santos, Laura Maria Vidal Nogueira, Regina Célia Gollner Zeitoune

**Affiliations:** 1 Universidade Federal do Rio de Janeiro, Escola de Enfermagem Anna Nery, Rio de Janeiro, RJ, Brazil.; 2 Secretaria Municipal de Saúde, Rio de Janeiro, RJ, Brazil.; 3 Instituto Nacional do Câncer, Rio de Janeiro, RJ, Brazil.; 4 Universidade do Estado do Pará, Escola de Enfermagem Magalhães Barata, Belém, PA, Brazil.

**Keywords:** Latent Tuberculosis, Meta-Analysis, Mycobacterium tuberculosis, Prevalence, Students, Systematic Review, Tuberculose Latente, Metanálise, Mycobacterium tuberculosis, Prevalência, Estudantes, Revisão Sistemática, Tuberculosis Latente, Metaanálisis, Mycobacterium tuberculosis, Prevalencia, Estudiantes, Revisión Sistemática

## Abstract

**Objective:**

The aim of this study was to synthesize the evidence on the prevalence of latent *Mycobacterium tuberculosis* infection (LTBI) among undergraduate health care students.

**Methods:**

A systematic review of prevalence with meta-analysis was conducted. Prospective and retrospective cohorts and cross-sectional studies involving probable exposure to *M. tuberculosis* during undergraduate education, along with the tuberculin skin test (TST) or interferon-γ release assay (IGRA) for investigation of latent tuberculosis were searched. Searches were conducted in MEDLINE, CINAHL, EMBASE, LILACS, Scopus, and Web of Science databases. Independent reviewers were responsible for the selection and inclusion of studies. Data were extracted, critically appraised, and synthesized using the JBI approach. PRISMA was used to report the study.

**Results:**

Twenty-two studies were analyzed. The overall prevalence in healthcare undergraduate students was 12.53%.

**Conclusion:**

The prevalence of LTBI in undergraduate health students was high for such a highly educated population. Screening with TST and/or IGRA and chemoprophylaxis, when necessary, should be provided to undergraduate health students when in contact with respiratory symptomatic patients.

## RESUMO

**Objetivo:**

O objetivo deste estudo foi sintetizar as evidências sobre a prevalência de infecção de Mycobacterium tuberculosis (ILTB) entre estudantes de graduação da área da saúde.

**Método:**

Foi realizada uma revisão sistemática de prevalência com metanálise. Coortes prospectivas e retrospectivas e estudos transversais envolvendo provável exposição a M. tuberculosis durante a graduação, juntamente com o teste tuberculínico (TT) ou ensaio de liberação de interferon-γ (IGRA) para investigação de tuberculose latente foram pesquisados. As buscas foram realizadas nas bases de dados MEDLINE, CINAHL, EMBASE, LILACS, Scopus e Web of Science. Revisores independentes foram responsáveis pela seleção e inclusão dos estudos. Os dados foram extraídos, avaliados criticamente e sintetizados utilizando a abordagem JBI. PRISMA foi usado para relatar o estudo.

**Resultados:**

Vinte e dois estudos foram analisados. A prevalência geral em estudantes de graduação da área da saúde foi de 12,53%.

**Conclusão:**

A prevalência de ILTB em estudantes de graduação em saúde foi alta para uma população com alto nível de escolaridade. Triagem com TT e/ou IGRA e quimioprofilaxia, quando necessária, deve ser fornecida aos estudantes de graduação da área da saúde quando em contato com pacientes sintomáticos respiratórios.

## RESUMEN

**Objetivo:**

El objetivo de este estudio fue sintetizar la evidencia sobre la prevalencia de infección latente por *Mycobacterium tuberculosis* (ILTB) entre estudiantes universitarios de la salud.

**Métodos:**

Se realizó una revisión sistemática de la prevalencia con metanálisis. Cohortes prospectivas y retrospectivas y estudios transversales que involucran exposición probable a *M tuberculosis* durante la educación universitaria, junto con la prueba cutánea de tuberculina (TST) o el ensayo de liberación de interferón-γ (IGRA) para la investigación de tuberculosis latente. Las búsquedas se realizaron en las bases de datos MEDLINE, CINAHL, EMBASE, LILACS, Scopus y Web of Science. Revisores independientes fueron responsables de la selección e inclusión de los estudios. Los datos se extrajeron, se evaluaron críticamente y se sintetizaron utilizando el enfoque JBI. Se utilizó PRISMA para informar el estudio.

**Resultados:**

Se analizaron veintidós estudios. La prevalencia global en estudiantes universitarios en salud fue del 12,53%.

**Conclusión:**

La prevalencia de LTBI en estudiantes universitarios de salud fue alta para una población con un nivel educativo tan alto. Se debe proporcionar tamizaje con TST y/o IGRA y quimioprofilaxis, cuando sea necesario, a los estudiantes universitarios en salud cuando estén en contacto con pacientes sintomáticos respiratorios.

## INTRODUCTION

Tuberculosis is an infectious disease, caused by the bacterium *Mycobacterium tuberculosis*, ranked among the top ten causes of death worldwide. Moreover, it is expected to be the second leading cause of death from a single infectious agent, until the COVID-19 pandemic^(1)^. Although tuberculosis is treatable, about 10 million new cases were reported globally in 2021, and almost 1.6 million people died from the disease, even with a reduction in notifications during the pandemic scenario^(1)^.

Tuberculosis primarily affects the lungs, and its contagion occurs through inhalation of droplets released by patients with active infection^(2)^. The risk of infection is directly related to the intensity and time of exposure to a bacilliferous person^(3)^. When the bacteria are inhaled, the immune system develops defense mechanisms through macrophages, producing granulomas. In most people (about 90%), the infectious process is maintained with bacterial replication in equilibrium and latent infection, called latent *M. tuberculosis* infection or latent tuberculosis infection (LTBI)^(2)^.

While infectious expression in LTBI is not induced, the body continues to generate an ongoing immune response to tuberculosis, with a 10% probability of progression to active tuberculosis^(4)^. Therefore, the World Health Organization (WHO) ranks the detection and prevention of LTBI as a crucial strategic component to prevent active tuberculosis^(4)^.

Global strategies and guidelines outline the importance of LTBI screening and treatment associated with early diagnostic testing to prevent new cases^(5)^. As no direct diagnostic methods of LTBI currently exist, T-cell response diagnostics are used, which are tuberculin skin test (TST) and interferon-gamma release assays (IGRA)^(4)^.

The TST and IGRA are the most relevant tests for identifying LTBI because, although there is no diagnosis for active tuberculosis, they indicate whether the individual has had contact with *M. tuberculosis* ^(6)^. The TST induces a hypersensitivity reaction via an intradermal injection of a purified protein derivative (PPD), which contains antigens from *M. tuberculosis*, nontuberculous mycobacteria, and *Mycobacterium bovis* Bacillus Calmette-Guerin (BCG). IGRA are in vitro blood tests that measure the amount of interferon-gamma (IFN-γ) produced by CD4+ T lymphocytes or the number of IFN-γ-producing T cells following stimulation with specific ESAT-6 and CFP-10 antigens of *M. tuberculosis* ^(7)^, showing more specificity than TST^(3,8)^.

Healthcare workers are among the most vulnerable groups to LTBI, which also include the following populations: those living on the street, deprived of liberty, and living with HIV, as well as migrants and indigenous peoples^(1,9,10)^. Health care professionals who are exposed to patients with the active form of tuberculosis are at greater risk of acquiring and, consequently, transmitting the bacillus^(11)^. 

Undergraduate health students involved in clinical practice are also exposed to occupational risks similar to healthcare workers^(3)^. Nursing students at a Brazilian public university showed an estimated 36% prevalence of LTBI^(3)^. Exposure of undergraduate health students to *M. tuberculosis* in high-prevalence settings is almost inevitable, as their training must take place in healthcare facilities with a high risk of infection^(12)^.

Therefore, the aim of this study was to synthesize the evidence on the prevalence of latent tuberculosis infection among undergraduate health students. The research question that guided the study was: What is the prevalence of latent tuberculosis infection among undergraduate health students?

## METHODS

This is a systematic review of prevalence, developed according to the Joanna Briggs Institute (JBI) recommendations^(13)^. The protocol was registered in the International Prospective Register of Systematic Reviews (PROSPERO) under the identification number CRD42020190716. The final report was constructed according to the PRISMA recommendations^(14)^.

This review included observational studies published in English, Portuguese, or Spanish, involving probable exposure to *M. tuberculosis* during undergraduate health education (academic activities and interaction with other students), along with the use of TST and/or IGRA to diagnose latent tuberculosis.

Studies involving the participation of students working in health services concurrently with those diagnosed with tuberculosis before joining the undergraduate course were excluded.

### Information Sources and Search Strategies

The search for the articles was performed in the CINAHL, EMBASE, LILACS, and MEDLINE via PubMed, Scopus, and Web of Science databases. The first search took place in September 2021 and the last search update took place in November 2022. The search strategies are available at the supplementary material Chart 1.

### Study Selection

Subsequent to the searches on the information sources, duplicates were removed. Titles and abstracts were independently reviewed by three reviewers based on the eligibility criteria, and then the entire texts were analyzed by the same group of reviewers. Mendeley software was used to manage the references. All studies excluded in the text-screening phase were characterized regarding the reasons for exclusion.

### Data Extraction

Data such as citation, study design, methods, country, subject, setting, year of data collection, participant characteristics, method of outcome measurement, and LTBI prevalence were independently extracted by two reviewers using a data extraction form created exclusively for this study. This form was based on the Joanna Briggs Institute (JBI) template for systematic reviews of prevalence and incidence^(13)^. Disagreements were discussed to reach consensus, but when this was not possible, a third, more experienced reviewer was activated for final decision.

### Risk of Bias and Certainty of Evidence Assessment

The GRADE – Grading of Recommendations Assessment, Development, and Evaluation^(15)^ was used to analyze the quality of the studies^(16)^. Methodological limitations in the study design and execution of methodological steps were assessed by means of the Joanna Briggs Institute checklists used for analytical cross-sectional studies^(17)^, prevalence studies^(18)^, and cohort studies^(18)^. Disagreements were resolved by consensus.

Heterogeneity was assessed using the I^2^ and X^2^ tests. A value greater than 50% was considered an indicator of substantial heterogeneity among studies. The level of statistical significance was fixed at 0.05 (p > 0.05). Indirectness and imprecision were evaluated with caution. Publication bias was assessed by means of funnel plots^(19)^, using the R x64 4.0.0 software, via RStudio Desktop.

### Data Analysis and Synthesis

Qualitative synthesis was performed in narrative and tabular form. Quantitative synthesis was performed via OpenMeta[Analyst] software, using the logit transformed proportion method in the random model with confidence interval established at 95%.

## RESULTS

### Study Selection

**Figure 1:**
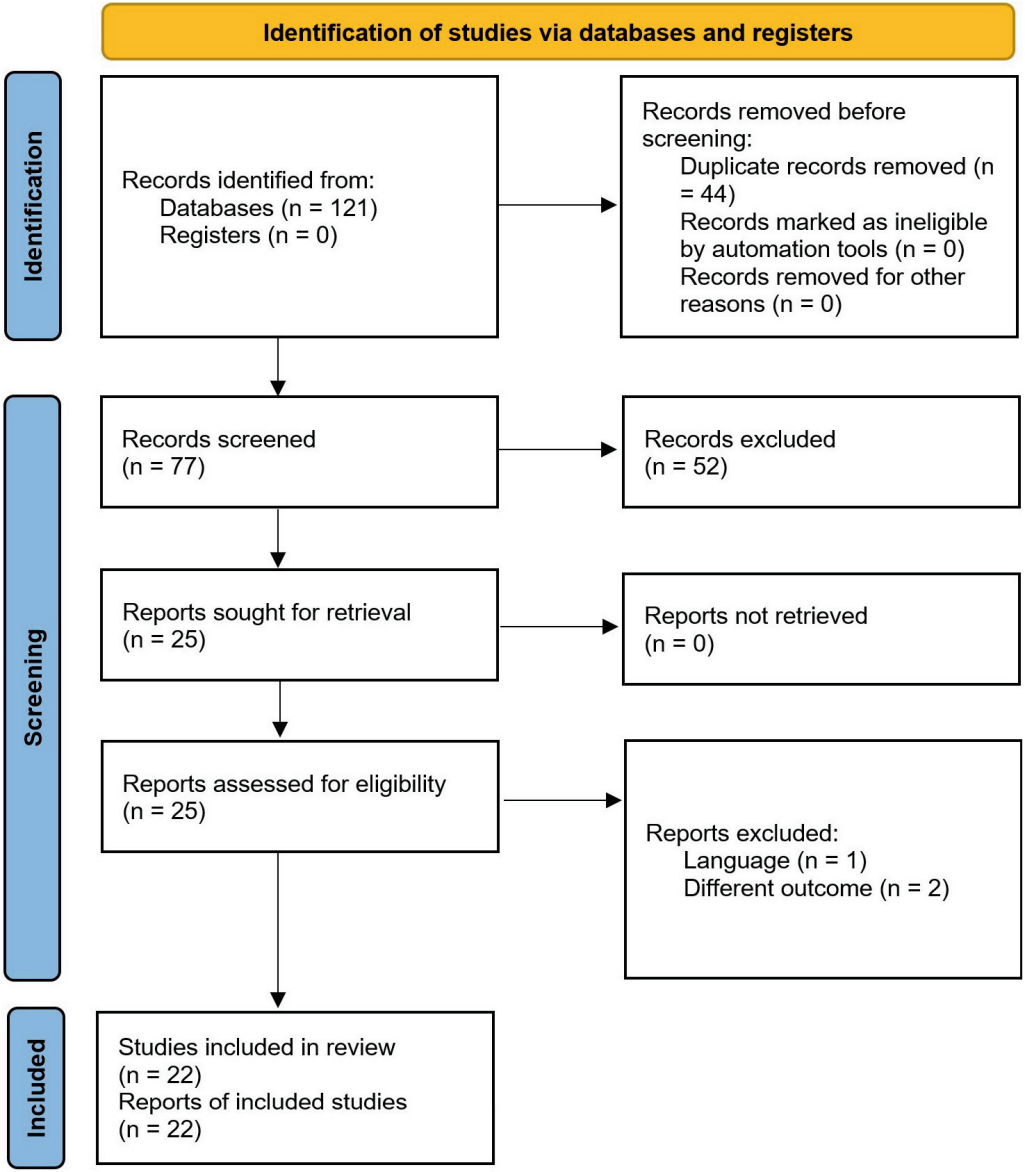
PRISMA flow diagram.

A total of 121 articles were found during the database searches. After removing 44 duplicates, 77 articles had their title and abstract screened. Twenty-five articles had their full-text screened, but only 22 studies were included in the sample. The reasons for exclusion of studies at the full-text screening phase was language (n = 1) and different outcome (n = 2). The PRISMA flow diagram is shown in Figure 1.

### Study Characteristics

Among the studies included in the analysis, 10 (45.45%) were published between 2015 and 2022, 10 (45.45%) from 2010 to 2014, 1 (4.54%) in 2006, and 1 (4.54%) in 2005. Fifteen (68.18%) were analytical cross-sectional studies, 3 (13.63%) were prospective cohort studies, 3 (13.63%) were retrospective cohort studies, and 1 (4.54%) was a descriptive cross-sectional study. The main characteristics of the studies are shown in Chart 2.

**Chart 2:** Main characteristics of the studies.

Study ID	Study type	Country	Undergraduate course	Diagnostic cut-off point	Inclusion criteria	Exclusion criteria
A1^(20)^	Cross-sectional analytic study	Italy	Undergraduate and graduate medical students	TST: ≥10 mm; IGRA: ≥ 0.35 IU	Asymptomatic undergraduate and graduate students who provided informed consent	Previous positive reaction to the Mantoux test or QTF-IT; loss to follow-up due to missed appointments for data collection, such as the TST test, quantiferon analysis, and the clinical record; inaccurate data provided; and refusal to sign the terms of free and informed consent
A2^(21)^	Cross-sectional analytic study	Italy	Medical, nursing, pediatric and obstetrical nursing	TST: ≥10 mm; IGRA: not informed	Students in the last three years of medical school (clinical practice) and all the first-year students in the nursing school, pediatric nursing, and obstetrics	Students in the last three years of medical school
A3^(22)^	Cohort study	Peru	Nursing, medical, pharmacology, laboratory technicians, engineering, preclinical nutrition, and veterinary	TST: ≥10 mm	Health care students (Nursing, Medical, Pharmacy and Laboratory Technicians) and non-health care students (mostly Engineering, plus Preclinical Nutrition and Veterinary)	History of active TB, history of positive TST, diagnosis of human immunodeficiency virus (HIV) or cancer, and chronic immunosuppressive treatment
A4^(23)^	Analytical cross-sectional study	Mexico	Undergraduate nursing students	Not informed	All the first and eighth semester students who wished to participate and signed the Free and Informed Consent Form	History of pulmonary or extra pulmonary tuberculosis; HIV infection; history of cancer, connective tissue disease, liver or kidney disease, those receiving chronic treatment with immunosuppressants, and pregnant women
A5^(24)^	Analytical cross-sectional study	Italy	Nursing and pediatric nursing students	TST: ≥10 mm; IGRA: ≥ 0.35 IU	All nursing and pediatric nursing students, attending 1st and 3rd years, were actively recruited to screen for TB risk assessment	Not informed
A6^(25)^	Analytical cross-sectional study	Venezuela	Dentistry	TST: ≥10 mm (students with induration > 15 mm were assessed for active TB)	Students in the 3rd, 4th, and 5th year of the Universidad Central de Venezuela School of Dentistry in 2007 who voluntarily agreed to participate in the study	History of pulmonary or extra pulmonary tuberculosis; HIV infection; history of cancer, connective tissue diseases, liver or kidney disease, and those who have received chronic treatment with immunosuppressants
A7^(26)^	Analytical cross-sectional study	Italy	Undergraduate medical and surgical students and health professions	TST: ≥10 mm	Students of the Undergraduate Course of Medicine and Surgery and the Health Profession at the University of Parma	Not informed
A8^(27)^	Cohort study	India	Nursing	TST: ≥10 mm	All nursing students were prospectively approached for participation in a cohort study to assess the prevalence and risk factors for LTBI and the annual rate of TB infection	Not informed
A9^(28)^	Cohort study	Peru	Dentistry, nursing, medical technology, medical, education, science, health administration, and veterinary	TST: ≥10 mm	Career variables were established: – Clinical career: dentistry, nursing, medical technology, and medical – Non-clinical career: education, health sciences and administration, veterinary	Students with a positive TST at the beginning of the study were excluded from the TST conversion analysis
A10^(29)^	Cohort study	India	Nursing	TST: ≥10 mm	All nursing students enrolled in 2007 in the different undergraduate and graduate programs	Students with a prior history of active TB were excluded from the study
A11^(30)^	Analytical cross-sectional study	Peru	Medicine	TST: ≥10 mm	Medical school students from the second to the seventh year of study	Not informed
A12^(31)^	Analytical cross-sectional study	Mexico	Dentistry	Not informed	Students of the Faculty of Dentistry	Not informed
A13^(32)^	Analytical cross-sectional study	Ethiopia	Physicians and paramedics	TST: ≥10 mm;	A13	Analytical cross-sectional study
A14^(33)^	Analytical cross-sectional study	Namibia	Not informed	Not informed	Not informed	Not informed
A15^(34)^	Cohort study	Saudi Arabia	Medical, dentistry, nursing, pharmacy, physiotherapy, laboratory sciences, and nutrition	TST: ≥10 mm	Undergraduate students who completed clinical training at the following colleges from 2010 to 2017: medicine, dentistry, nursing, pharmacy, and applied medical sciences (including physical therapy, laboratory sciences, and nutrition). Students tested for TB with the TST before and after clinical training as undergraduate students were included	Students with active TB were excluded
A16^(35)^	Analytical cross-sectional study	Malaysia	Medical	IGRA: ≥ 0.35 IU	1st and 5th year medical students of the Faculty of Medicine and Health Sciences, University Putra Malaysia	Active TB, treated for TB, pregnant, with acute infection, or taking immunosuppressive drugs
A17^(36)^	Analytical cross-sectional study	Brazil	Medical	TST: ≥10 mm	Undergraduate medical students from March 2002 to September 2003	Active TB and those who did not want to participate
A18^(37)^	Cohort study	Peru	Medical	TST: ≥10 mm	Students enrolled in medical school at the time of data collection and consenting to the use of their clinical information for analysis	Those who had a positive reading (positive TST) at the beginning of the study were excluded
A19^(38)^	Analytical cross-sectional study	Brazil	Medical	TST: ≥5 mm	Medical students at the Universidade do Vale do Sapucaí in 2010	Not informed
A20^(39)^	Analytical cross-sectional study	India	MBBS, Bachelor of Science (nursing), and engineering	TST: ≥10 mm	Students admitted to MBBS, Bachelor of Science (nursing) and engineering courses from 2011 to 2014	Those on treatment for active tuberculosis, with a previous history of the disease, and immunocompromised in whom the interpretation of the TST could be falsely negative
A21^(40)^	Analytical cross-sectional study	Brazil	Nursing and medicine	TST: ≥10 mm	Undergraduate nursing and medical students	Not informed
A22^(41)^	Descriptive cross-sectional study	Thailand	Medicine	TST: ≥10 mm IGRA: ≥ 0.35 IU/ml	Fourth-year and sixth-year medical students	Medical students who had a history of TB, were pregnant, received systemic immunosuppressive therapy, or aged < 18 years

The studies were conducted in Italy (n = 4; 18.18%), Peru (n = 4; 18.18%), Brazil (n = 3; 13.63%), India (n = 3; 13.63%), Mexico (n = 2; 9.09%), Ethiopia (n = 1; 4.54%), Malaysia (n = 1; 4.54%), Namibia (n = 1; 4.54%), Saudi Arabia (n = 1; 4.54%), Thailand (n = 1; 4.54%), and Venezuela (n = 1; 4.54%). Therefore, 3 (13.63%) studies were conducted in high-burden tuberculosis, HIV-associated tuberculosis, and rifampicin-resistant/multidrug-resistant tuberculosis countries; 4 (18.18%) in high-burden rifampicin-resistant/multidrug-resistant tuberculosis countries; and 6 (27.27%) in high-burden tuberculosis and HIV-associated tuberculosis countries.

### Risk of Bias in the Studies

The results of using the Joanna Briggs Institute checklists for cohort studies and analytical cross-sectional studies are available at the supplementary material tables 2 and 3 (https://osf.io/download/93ucz/). An important risk of bias was neglecting the confounding variables in both cohort studies and analytical cross-sectional studies. History of tuberculosis diagnosis, of direct contact with tuberculosis patients, of working in health care, and of presence of immunosuppressive condition or treatment and BCG scar are the main confounding variables, and few studies (A5^(24)^, A8^(27)^, A10^(29)^, and A22^(41)^) accounted for them adequately.

### Qualitative Synthesis

The prevalence of LTBI among undergraduate health students ranged from 1.1% (95% CI 0.7–1.6%) in a study conducted in Italy^(24)^ to 50.23% (95% CI 45.43–55.02%) in another study from India^(29)^. Table 1 presents the main results of the individual studies, including sample size, frequency of LTBI diagnoses, prevalence expressed as a percentage, the country in which the study was conducted, and whether that country has high tuberculosis, tuberculosis/HIV, and/or MDR/RR- tuberculosis burden, according to the World Health Organization^(42)^.

**Table 1:** Prevalence of latent *Mycobacterium tuberculosis* infection among undergraduate health students identified in the individual studies.

Study ID	Events	Sample	Prevalence (%)	Country	High-burden country
A1^(20)^	23	2082	1.10	Italy	No
A5^(24)^	20	1564	1.28	Italy	No
A2^(21)^	10	733	1.36	Italy	No
A7^(26)^	17	513	3.31	Italy	No
A22^(41)^	6	158	3.79	Thailand	TB and TB/HIV
A16^(35)^	6	143	4.20	Malaysia	No
A15^(34)^	108	1822	5.93	Saudi Arabia	No
A17^(36)^	71	1032	6.88	Brazil	TB and TB/HIV
A14^(33)^	28	290	9.66	Namibia	TB and TB/HIV
A9^(28)^	527	3994	13.19	Peru	MDR/RR-TB
A18^(37)^	79	505	15.64	Peru	MDR/RR-TB
A3^(22)^	164	944	17.37	Peru	MDR/RR-TB
A11^(30)^	98	548	17.88	Peru	MDR/RR-TB
A6^(25)^	23	127	18.11	Venezuela	No
A4^(23)^	15	70	21.43	Mexico	No
A21^(40)^	54	225	24.00	Brazil	TB and TB/HIV
A19^(38)^	34	120	28.33	Brazil	TB and TB/HIV
A20^(39)^	155	522	29.69	India	TB, TB/HIV and MDR/RR-TB
A12^(31)^	70	200	35.00	Mexico	No
A8^(27)^	339	755	44.90	India	TB, TB/HIV and MDR/RR-TB
A13^(32)^	50	107	46.73	Ethiopia	TB and TB/HIV
A10^(29)^	219	436	50.23	India	TB, TB/HIV and MDR/RR-TB

Note: TB, Tuberculosis; TB/HIV, Human Immunodeficiency Virus and Tuberculosis co-infection; MDR/RR-TB, Multidrug Resistant/Rifampicin Resistant Tuberculosis.

Nine studies have shown the prevalence of LTBI from both among undergraduate health students in the pre-clinical phase (before entering health care facilities) or undergraduate students from other nonclinical areas (those that do not involve entering health care facilities) and among undergraduate health students in the clinical phase (inside health care facilities). Data of sample sizes, frequency of LTBI diagnoses, and prevalence expressed as percentages in the pre-clinical, pre-non-clinical, and clinical phases, and whether the country in which the study was conducted has high tuberculosis, tuberculosis/HIV, and/or MDR/RR-tuberculosis burden are available at the supplementary material table 4 (https://osf.io/download/93ucz/).

The presence of a BCG vaccine scar was evaluated in 3 studies. One of these studies^(35)^ was conducted at the University of Putra Medical School in Malaysia, where 85.3% and 97.3% of 1st year and 5th year students had BCG vaccine scarring, respectively. Another study, conducted at the Universidade Autônoma de Querétaro in Mexico^(23)^, found that 100% of nursing students had a BCG scar. A study conducted at Addis Ababa University, in Ethiopia^(32)^, identified that 44.9% of medical students had BCG vaccine scar.

Two studies evaluated the likelihood of tuberculin skin test (TST) positive detection in students with BCG vaccine scars, especially those who received the booster dose of the vaccine, when compared to those who were not vaccinated. Both comparisons showed no statistically significant differences^(26,28)^. Another study found no statistically significant association between TST positivity and age, sex, or BCG vaccination in medical and nursing students from Italy^(21)^. Two other studies – one conducted with medical students in Brazil^(36)^ and another with nursing students in India^(29)^ – showed that the BCG scar was statistically significantly associated with positive TST result. Research conducted in Italy, with medical students, showed no statistically significant association between the presence of a BCG vaccine scar, or originating from countries with a high tuberculosis burden and a positive TST result^(26)^. Another study, whose participants were undergraduate and graduate health students in Italy, found that 17.0% of graduate medical students received BCG vaccination compared to 0.24% of undergraduate students^(20)^.

Four studies showed reduced diagnoses of LTBI when, in addition to TST, confirmation was performed with the IGRA via Quantiferon-Tuberculosis test (QFT)^(20,21,24,32)^. 

### Quantitative Syntheses

**Figure 2:**
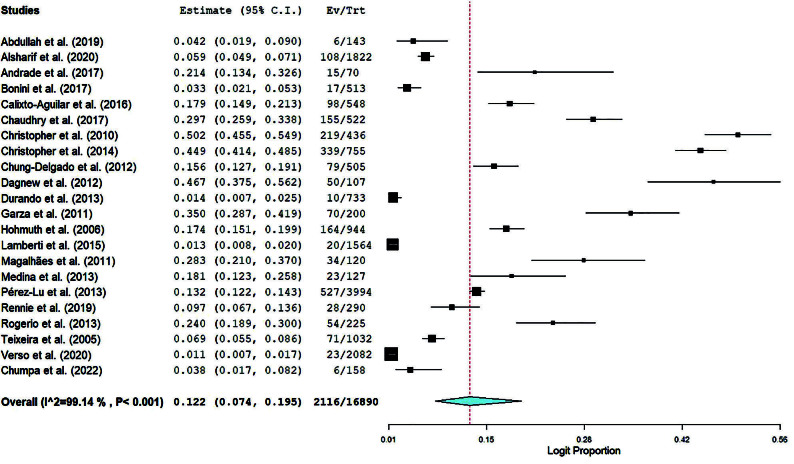
Forest plot of overall LTBI prevalence among undergraduate health students.

Figure 2 presents the forest plot that summarizes the overall prevalence of LTBI among undergraduate health students. Other forest plots of the additional sub-group analysis are available at the supplementary material figures 1–4 (https://osf.io/download/93ucz/).

### Quality of the Evidence

The certainty of the body of evidence was very low for the main outcome, downgraded from high due to risk of bias, heterogeneity, and publication bias. The certainty of the body of evidence was low for all subgroup analysis due to risk of bias, heterogeneity, and publication bias.

The funnel plots^(19)^ of the main meta-analysis and of the additional sub-group analysis are available at the supplementary material figures 5–9 (https://osf.io/download/93ucz/). All tests indicated asymmetries typical of publication bias, although all meta-analyses, except the one with all the included studies, showed small number of studies, and the low proportion of the outcomes may have overestimated the asymmetries^(43)^.

## DISCUSSION

This systematic review showed that, similar to health professionals, students in health professions are also at significant risk of having been diagnosed with LTBI. In a population from which is expected knowledge regarding tuberculosis transmission and prevention as well as access to personal protective equipment (PPE), the overall prevalence in healthcare undergraduate students was 12.53%, while the overall prevalence across all populations ranged from 17.4% to 24.2%^(44)^. The main limitation of this systematic review is the language restriction.

In order to minimize the risks inherent to occupational exposure to which health students are subjected, the importance of educational programs on the proper use of PPE is relevant, as its proper use effectively prevents transmission by contact and droplets^(45,46,47)^. PPE is put on and taken off incorrectly by healthcare professionals very often^(48)^. Therefore, it is suggested that a significant number of infections could be avoided by professionals and students using individual protective measures. Education and improved awareness of the risks of acquiring tuberculosis are required to reduce the possibility of infection, with tutors expected to instruct their students on the correct management practices and use of PPE^(49)^.

A study conducted in India demonstrated that nursing and medical students are 2.54 times more likely to suffer from ILTB than non-healthcare undergraduate students, and there is a statistically significant increase in positivity for TST, as the duration of exposure to clinical practices increases^(39)^. During the early (pre-clinical) years, minimal exposure to clinical facilities occurs. However, students in later undergraduate terms are at higher risk for LTBI, as they are expected to spend more time in hospital settings to experience closer contact with patients^(35)^, increasing their risk of exposure to tuberculosis cases.

Regions of high tuberculosis incidence and prevalence have a high possibility of contact with tuberculosis patients, although it is more difficult to determine whether the exposure was actually occupational or communal. In countries with a high burden of tuberculosis, such as India, inadequate or no screening of outpatients with proven or suspected tuberculosis, inadequate ventilation, and overcrowding are observed in settings in which care is provided^(29)^. In this setting, healthcare professionals and undergraduate students provide care while maintaining close contact with patients with infectious tuberculosis^(29)^. 

A study conducted in Brazil^(40)^ showed that the time allocated to practical teaching of tuberculosis management ranged between 10 and 20 hours and occurred mostly in Primary Health Care services. Further studies in different countries would help determine in which health services undergraduate health students are most vulnerable in clinical practice.

Biological and social factors have a direct impact on the vulnerability to tuberculosis, such as malnutrition, age group, HIV infection, unhealthy housing, high population density, difficult access to health services, inadequate working conditions, among others^(50)^. In addition to these factors, this study, as well as others^(21,35)^, has shown that exposure during clinical practice in undergraduate health courses also increases the risk of exposure to *M. tuberculosis*, and consequently, LTBI. Thus, it is recommended that TST be conducted among undergraduate health students, both at baseline and throughout the course, to screen for LTBI as part of a tuberculosis screening program, which would include periodic clinical assessment.

The primary limitation of this review is the comprehensiveness of the searches. The inclusion criteria were restricted to the English, Portuguese, and Spanish languages, so relevant studies published in other languages may exist and were not included. Furthermore, despite consulting five of the most relevant health databases, relevant documents published in journals indexed in other databases may exist and were not included. Future research investigating the effectiveness of annual screening with TST and/or IGRA in health students, as well as chemoprophylaxis in cases of LTBI in this population, should be conducted.

## CONCLUSION

The prevalence of LTBI in undergraduate health students was 12.53%, a significant and elevated proportion for a highly educated population, which is expected to have access to and adequate instruction in the use of PPE. This systematic review has contributed to evidence that, in addition to professionals, health care students are also a vulnerable group to LTBI. Annual screening for tuberculosis, including the TST and/or IGRA, in undergraduate health students from the beginning of their courses, can both facilitate the early diagnosis of LTBI, anticipate chemoprophylaxis, and prevent the manifestation of tuberculosis in this population.
